# Psychometric Indicators of the Pro-environmental Attitudes' Questionnaire: Colombian Version

**DOI:** 10.3389/fpsyg.2022.886769

**Published:** 2022-07-12

**Authors:** Willian Sierra Barón, Alba Lucia Meneses Baez

**Affiliations:** ^1^Faculty of Social Sciences and Humanities, Universidad Surcolombiana, Neiva, Colombia; ^2^Faculty of Psychology, Universidad Católica de Colombia, Bogotá, Colombia

**Keywords:** Colombian workers, instrument reliability, instrument validity, Rasch model, pro-environmentally attitudes, PEAQ

## Abstract

The detrimental effect of human behavior on the environment is undeniable. Attitudes are recognized as a predictor of the pro-environmental behavior; therefore, having good quality tools in Colombia to measure them is strategic to assess interventions. This study aims to establish psychometric indicators for the pro-environmental attitudes questionnaire (PEAQ) Colombian version to a sample of 415 volunteers (53% women and 47% men) aged 18–70 years (M = 40.28; SD = 14.06). We used the 28-item PEAQ already linguistically adapted for Colombia. We applied the following questionnaires: Environmental awareness (EA) (11 items), environmental values (EV) (4 items), and the pro-environmental at work questionnaire (PEWQ) (31 items). We used a one-parameter Rasch model and Winsteps program to assess the PEAQ's one-dimensionality and item statistics by gender, and estimated Spearman's rho coefficient between the PEAQ scores and the scales for concurrent validity. The PEAQ in this study has 24 items because 4 items did not fit into the Rasch model criteria. Its one-dimensionality was supported by an explained variance (43%) and the first residual variance (12%). The coefficients, α = 0.95 and Ω = 0.95; Rasch for persons = 0.90; and Rasch for items = 0.95. The correlation between the PEAQ and the EC, EV, and PEWQ scales were Spearman's rho coefficient = 0.859 (*p* ≤ 0.001), 0.795 (*p* ≤ 0.001), and 0.885 (*p* ≤ 0.001), respectively. Thus, the PEAQ Colombian version's psychometric indicators support it as a valid and reliable instrument to measure pro-environmental attitudes in this country.

## Introduction

The global warming produced by mankind threatens the survival of the planet, including humans (IPCC, 2020); proof of this is the increasing scarcity of water, the public health problems such as air pollution and lack of drinking water as well as the prospect that carbon dioxide (CO_2_) emissions will continue going up. The developed countries contribute significantly to the greenhouse effect due to their high industrialization that causes, between others, a high rate of CO_2_ emission and the consequences of this contamination impact the whole world (Mott et al., 2021). On the other hand, there are individuals' behaviors that contribute significantly to the greenhouse effect such as high electric energy consumption, low recycling, and the use of toxic substances on daily activities (International Energy Agency, 2016; IPCC, 2020). Additionally, the developing countries are more affected in all these aspects due to their low pro-environmental investment (Dhrifi et al., 2020; Ahmad et al., 2021).

Gifford et al. (2011) point out the need to investigate the relationships between the physical environment and factors such as the development of people's daily activity, feelings, attitudes, and behaviors. Therefore, psychology can help to improve the pro-environmental behavior by studying the environmental attitudes that can contribute to the emergence of ecological behavior.

According to Félonneau and Becker (2008), attitudes toward the environment are beliefs, affections, and intentions of individuals regarding the development of activities and decisions that favor a sustainable planet. Consequently, the study of pro-environmental attitudes which are considered predictors of pro-environmental behavior can prospectively contribute to the systematic design and evaluation of the pro-environmental behavior intervention programs as well as feedback into national environmental policy that could positively impact the consumer behaviors (Paillé et al., 2013; Robertson and Barling, 2015; Zhang, 2019).

Although the pro-environmental attitude is not the only important variable in improving the world's environmental conditions, contributing to its development and consolidation can increase the likelihood that consumers will develop environmentally friendly behaviors that reduce the effects of global warming (Robertson and Barling, 2015; Berger and Wyss, 2021).

Measuring a phenomenon is a step toward its characterization; this includes establishing the interactions between its components and evidencing their relative weights, information that contributes to its understanding, which is fundamental for the technological development that may modify its course in the future (Wilson, 2013).

Colombia currently has policies and legislation on environmental regulation developed from the country's 1991 Political Constitution (Political Constitution of Colombia, 1991), which stipulates the right of all citizens to have a healthy environment (article 79) and to prioritize the environment over the right to economic freedom, among others (article 333). The country has a National Environmental System under the direction of the Ministry of Environment and an environmental protection plan (Development Plan, 2018–2022).

Despite these policies, regulations, and an environmental management system, there are still no programs in the country aimed at promoting and improving the sustainable environmental practices, especially those that impact human behavior (Bugdol et al., 2021), and there is no evidence on instruments for measuring the pro-environmental attitudes with psychometric properties appropriate to the context, which indicates that Colombia lacks measurement tools for this construct, which would allow having baselines for the design of interventions as well as monitoring these actions that could contribute to the development of this type of human behavior.

## Objective

To assess the psychometric properties of the pro-environmental attitudes questionnaire (PEAQ) by Félonneau and Becker (2008) on the Colombian population.

## Study Type

This is a cross-sectional study according to the definition of Kesmodel (2018).

## Method

### Participants

The sample consisted of 415 volunteer workers (53% women and 47% men). All participants met the following inclusion criteria: More than 18 years of age, currently an employee of an organization in the geographic territory of Huila in Colombia, and performing functions inside the organization's infrastructure instead of outside of the campus, e.g., on field work. The sample was not randomly selected. The participants' organizations were two higher education institutions (public and private), and a local government organization.

The participants reported a mean age of 40.28 years (SD = 14.06); 51.1% were married, 26.5% were single; 47.4% had received undergraduate or graduate training; 73.7% belonged to socioeconomic stratum 1, 2, and 3; contractually, only 20% reported being employed for an indefinite term. A total of 29.2% worked in positions associated with secretaries or administrative assistants (Table 1).

**Table 1:** Participants' socio-demographic characteristics (*N* = 415).

**Variables**	**Subcategories** **(M; SD)**	**Frequency**	**Percentage**
Age	(40.28; 14.06)	–	–
	Women (40.57; 13.93)	–	–
	Men (39.95; 14.25)	–	–
Gender	Men	220	53
	Women	195	47
Civil status	Married	141	34
	Free union	71	17.1
	Divorced	63	15.2
	Widower	30	7.2
	Single	110	26.5
Education level	Elementary	34	8.2
	Secondary	43	10.4
	Technician (2 years of technical studies)	67	16.1
	Technologist (3 years of technical and theoretical studies)	74	17.8
	Undergraduate	113	27.2
	Graduate	84	20.2
Socioeconomic level	1	51	12.3
	2	140	33.7
	3	111	27.7
	4	54	13
	5	31	7.5
	6	28	6.7
Type of contract	Indefinite term	83	20
	Fixed-term	104	25.1
	Service contractor	92	22.2
	Occasional job	35	8.4
	Apprenticeship	64	15.4
	Other	32	7.7
Position	Apprentice, intern	48	11.6
	Consultant	17	4.1
	Professional	37	8.9
	Director, manager, area or office coordinator	26	6.3
	Teacher/Instructor	44	10.6
	Support, administrative and specialized professional	48	11.6
	Secretary, assistant/administrative	121	29.2
	General services, operator driver	38	9.2
	Salesperson	36	8.7

### Instruments

Pro-environmental attitudes questionnaire (PEAQ) (Félonneau and Becker, 2008), which consists of 28 Likert-scale type items (1 = never and 4 = always); in its original version, the instrument shows Cronbach's alpha coefficient, α = 0.93. For the linguistic adaptation of the PEAQ, two psychologists, one specialized in organizational psychology and the other in psychometrics, with Spanish as native language and over 2 years residing in Canada, translated independently the questionnaire from English to Spanish. Later, the researchers met with them to resolve discrepancies obtaining a unified version of the test. An official translator residing in Colombia over 30 years that has English as native language translated this version to English to accomplish backward–forward translation. Then, the three translators, the researchers, and a bilingual psychologist specialized in organizational psychology, with Spanish as native language residing in Huila, met to resolve discrepancies and to stablish conceptual, cultural, and linguistic equivalence of the questionnaire (Hambleton and Zenisky, 2011). With the resulting version, the researchers conducted 10 cognitive interviews with potential users that were ask to identify the difficulties to understand all parts of the questionnaire, as well as their pertinence to their context, and to make suggestions to correct them. The results showed that users had problems in understanding the Likert scale and some items. Based on their suggestions, the scores were changed to a frequency scale and 3 items were adjusted, this new version was used in cognitive interviews to 10 new potential users and based on the results one item was adjusted. The resulting version was used in a pilot study with 450 workers of 4 different organizations in the Huila territory (52.4% females, 47.6% males). As a result of the pilot study, we reduced the categories to 4, since there was not enough distance between categories 1 and 2, and we adjusted 3 items. The Cronbach's alpha in that pilot study was α = 0.95.

The environmental awareness (EA) scale (Blok et al., 2015) measures the knowledge the workers have about the effects of human behavior on the environment, and about the effects of the environment on human beings, including the awareness of environmental issues. This knowledge is based on the understanding of the environment and the workers' ability to act responsibly providing solutions to environmental issues (Grob, 1995). It includes 11 Likert-scale type items (1 = strongly disagree; 5 = strongly agree) and shows α = 0. 85 in the original version, and in the Colombian version, α = 0.89 CI 95% [0.87, 0.90] (Sierra-Barón and Meneses, Forthcoming)^1^.

The environmental values (EV) scale (Blok et al., 2015) measures the fundamental beliefs about nature and the environmental protection organized according to the individual importance, which guide people's pro-environmental attitudes and behaviors (Schwartz, 1994; Stern et al., 1999). It includes 4 Likert-scale type items [(1 = strongly disagree e; 5 = strongly agree) and shows α = 85 in the Dutch version (Blok et al., 2015), and in the Colombian version, α = 0.83 CI 95% [0.79, 0.85]^1^].

The pro-environmental at work questionnaire (PEWQ) measures activities and behaviors of the employee in the workplace that seek to reduce the negative impact on the environment, which can be caused both by the activities of the organizations themselves and by employees in their personal activities, which consists of 31 Likert-scale type items shows α = 0.95 CI 95% (0.95, 0.96), elaborated from the proposals of Blok et al. (2015) and Wesselink et al. (2017) by Sierra-Barón and Meneses (Forthcoming)^1^.

Sociodemographic data questionnaire obtains data on the participants' date of birth, gender, marital status, educational level, socioeconomical level, and type of contract and position.

### Procedure

The researchers contacted the authorities of two institutions of higher education in the city of Neiva (Huila), one public and one private, and a public social service organization to request authorization to conduct the study. Once authorization was obtained, the researchers made a call by institutional mail with a schedule in each institution to explain the study, apply the informed consent, and the personal data authorization form. All workers who signed both documents were given the printed questionnaire to fill it out later along with the EA, EV, and PEWQ tests. Sometimes, it was applied in groups and other times individually. The participants did not receive any incentives for completing the questionnaires.

Data analysis was done as follows: Once the data had been collected and compiled, the authors analyzed them with the SPSS v.23.0 program and the Winsteps v.3.80.1.

First, the response omission rate for each questionnaire and the total response omission rate were estimated. The acceptability to determine the absence of systematic bias for missing data was that their percentage for variable were <10% and for person that they had not answered up to three questions. We took into account that the missing data were not focus in some variables.

Second, the demographic characteristics of the participants were obtained through descriptive statistical analyses (Table 1).

Third, we conducted normality tests for the PEAQ scores and the referential instruments scores (EA, EV, and PEWQ) using Kolmogorov–Smirnov normality test. Most of the scores did not exhibit normality; therefore, Spearman's rho coefficient was computed to estimate the existing correlations between these variables scores as evidence for concurrent validity having a criterion, *r* ≥ 0.45 (DeVon et al., 2007).

Fourth, the authors obtained evidence on the structure of the construct measured by the PEAQ, assessing the unidimensionality of the questionnaire using the one-parameter Rasch model and the program Winsteps v. 3.80.1.

The one-parameter Rasch evaluation model is a probabilistic mathematical model of item response theory (IRT) that meets the properties of linear scaling and interval measurement. In this model, a person with high ability will always have a higher probability of answering an item correctly or accepting it than a person with low ability, and a more difficult item will always have a lower probability of being answered correctly than an item with lower difficulty, regardless of the person's ability level (Conrad and Smith, 2004; Bond and Fox, 2015).

The assumption of the Rasch model is that a test measures a single construct when its items form a hierarchical continuum; its units of measurement are additive, providing estimates that support its validity, in general, this occurs when the data fit the model (Wilson, 2005; Bond and Fox, 2015).

In this study, we used the Rasch model for polytomous items, taking into account the ordered categorical responses in a Likert scale. This model estimates the probability that a respondent has the choice of selecting a category of response for a determined item; this estimation is made through the formula:


$$\begin{array}{l} \text{ln}\left[P_{nij}/{P_{ni}}_{\left(\text{j}-1\right)}\right]=B_{n}-D_{i}-F_{j} \end{array}$$

where *P*_nij_ is the probability of the respondent *n* to choose the category *j* of the item *i*, *P*__*ni*_(j−1)_ is the probability of the same respondent to score the category *j* – 1 of the item *i, B*_*n*_ is the measure of the skill of the respondent *n, D*_*i*_ if the difficulty of the item *i*, and *F*_*j*_ if the difficulty of the category *j*. The categories in this questionnaire are organized in steps in the measure scale (Wright and Masters, 1982). In our Likert scale; for example, *j* would be “always” and *j* – 1 would be “almost always.”

Unidimensionality evidence: According to Bond and Fox (2015), the continuum of item difficulty levels and people's ability is represented using the Rasch rule; in this rule, the positive and high values indicate higher item difficulty and the negative values indicate low difficulty. The items and people who present a level of difficulty or ability with a value lower than 0 logits in the Rasch rule present low difficulty or low level of ability, respectively, in contrast to the items and people whose level of difficulty or ability have values higher than zero; the farther the value is from zero and positive, the higher the level of difficulty of the item or the higher the ability of the people and it is expected that the level of difficulty of the items covers the totality of the abilities of the sample (Bond and Fox, 2015).

To evaluate the fit of the data to the Rasch model, the statistical indicators of near fit (infit) and far fit (outfit) are used, which help to establish the quality of each item and the fit to the model of the evaluated persons who answered those items; the first statistic is sensitive to the execution of the persons whose performance is closer to the difficulty level of the item and the second refers to the extreme values of the responses to the items. Both indicators handle the statistical form χ^2^ divided by its degrees of freedom with an expected value, 1, and a range between 0 and +∞; any value >1 signals a higher percentage of variation between the observed response patterns and those predicted by the model and a value <1 implies a lower percentage of variation in the observed response pattern than that predicted by the model (Linacre, 2012; Bond and Fox, 2015).

The item discrimination statistic indicates an item response variation level in relation to the person's skill level and shows the degree that an item was answered correctly by people with high skill level and incorrectly by those with a low skill level. Items are adequate with values more than or 0.7 (ETS, 2000, quoted by Pardo and Rocha, 2010).

In this study, it was taken as a criterion for infit and outfit statistics that the values obtained for each item are in the range between 0.7 and 1.3, and that the value of the biserial point correlation product moment (PTME) measure of each item be more than or 4, as an indicator that the items behave as parts of the measured construct (Wilson, 2005; Linacre, 2006).

In Rasch model, an instrument measures a single construct when the variance explained by the measure is more than or 20% and the percentage of the variance not explained by the first contrast is <20% (Reckase, 1979); and the eigenvalue is <3 (Linacre, 2006; Bond and Fox, 2015). In this study, we take as a criterion for the variance explained more than or 40, 2 times the minimum proposed by Reckase (1979), taking into account that Rasch model demands unidimensionality and we wanted to reduce the margin of having more than one dimension.

Fifth, the authors estimated the Rasch reliability indexes and the internal consistency coefficients Cronbach' alpha and omega. The Rasch model has reliability indexes for the people and items (Boone and Staver, 2020). These indexes are analog to Cronbach's alpha coefficient, their values are 0 ≤ α ≤ 1, and the criteria used is α ≥ 0.80 (Nunnally, 1978; Bond and Fox, 2015). Rasch model has other reliability indicators for items and persons called “separation measure” that points out the number of levels in standard error units, in which the item and person samples can be grouped together. To get at least more than one group, the criteria expected for the separation measure should be more than or 1.75 (Boone and Staver, 2020). Additionally, we estimated the omega coefficient for each scale using the SPSS v.23.0 program; since this coefficient exhibits greater stability, because it is estimated from the factorial weights, it does not depend on the number of items and does not require compliance with the tau equivalence principle (McDonald, 2011; Muñiz, 2018).

Sixth, following the recommendation of American Educational Research Association (AERA) et al. (2014), the authors estimated the differential item functioning (DIF) of the PEAQ in the subgroups of the variables gender and educational level to identify if there was any systematic bias in the test score. To assess measure's bias, the DIF is estimated by subgroups because the item difficulty level must remain invariant between them. A significant contrast DIF is one that has a ≅0.5 logit difference for all comparisons (*p* ≤ 0.05) when estimating the Mantel–Haenszel statistic that is approximately greater than or SD/2 of the people skill level (Bond and Fox, 2015). The Educational Testing Service (ETS) suggests that the values <0.43 are negligible; values more than or 0.43 logits are considered slight-to-moderate impact, and values more than 0.64 logits are moderate to large (Zwick et al., 1999).

## Results

We present evidence for PEAQ psychometrics indicators based on the scores obtained from 495 workers.

### Acceptability

The matrix of data collected from the scales and questionnaires applied showed a response omission rate of 1.42%.

### Pro-environmental Attitudes Questionnaire Unidimensionality Evidence

The pro-environmental attitudes questionnaire shows an explained variance of 47.4%, and a variance of the first residual of 4.3% with 1.67 eigenvalue; and the relationship between the two variances is >3–1. Additionally, the difficulty of items in logits for the questionnaire shows a range of −0.23 to + 0.34.

The PEAQ items values for infit and outfit statistics (Table 2) meet the Rasch model criteria (0.7–1.3), the error values for all items are ≅0, all items' PTME values are 0.61–0.70 and the item's discrimination index is >0.7 for all items (Table 2).

**Table 2:** Goodness-of-fit statistics Rasch model study PEAQ scale Colombian version (*N* = 415).

**Item**	**Difficulty**	**Error**	**Infit**	**Outfit**	**PTME**	**Discrimination index**
PEAQ3. El esforzarme por no usar productos de limpieza y fragancias para la casa, potencialmente toxicas contribuye a mantenerme saludable.	−0.23	0.06	1.06	1.28	0.64	0.73
PEAQ5. Reciclar mi basura es bueno para mi comunidad.	0.05	0.06	0.85	0.80	0.71	1.31
PEAQ6. Para mí es importante depositar siempre mi basura en los lugares establecidos para ello.	−0.1	0.06	0.91	0.86	0.70	1.20
PEAQ7. Considero importante cuidar mi consumo personal de agua a pesar de que las industrias la desperdician.	−0.03	0.06	1.04	1.00	0.67	1.03
PEAQ8 Creo que el consumo de productos orgánicos puede contribuir a la preservación del planeta.	0.02	0.06	0.87	0.81	0.71	1.35
PEAQ9. Mi deber como ciudadano es consumir menos energía como sea posible.	−0.18	0.06	1.08	1.09	0.68	1.00
PEAQ10. Mi deber como ciudadano es asegurarme de depositar la basura en el lugar indicado.	−0.02	0.06	0.84	0.81	0.70	1.17
PEAQ11. Una forma de respetarme a mí mismo es reciclar mi basura.	0.07	0.06	0.88	0.90	0.68	1.12
PEAQ12. Tomar el transporte público en lugar de usar mi carro es un comportamiento amigable con el ambiente.	0.14	0.06	0.94	0.98	0.65	0.96
PEAQ13. Es gratificante clasificar mi basura.	0.09	0.06	1.03	1.03	0.66	1.04
PEAQ14. Considero importante disminuir el uso de aerosoles.	0.05	0.06	0.86	0.81	0.70	1.27
PEAQ15. El uso reducido de aerosoles puede ayudar a preservar el planeta para las generaciones futuras.	−0.2	0.06	1.17	1.21	0.65	0.83
PEAQ16. Usar los aerosoles lo menos posible, ayuda a preservar mi salud.	−0.01	0.06	0.86	1.00	0.69	1.17
PEAQ17. Tomar el transporte público tiene varias ventajas (económicas, ambientales, de tiempo).	−0.09	0.06	1.15	1.21	0.63	0.74
PEAQ18. Consumir productos orgánicos beneficia mi salud.	0.34	0.06	1.03	1.07	0.65	1.07
PEAQ19. Reciclar mi basura contribuye a proteger el medio ambiente.	0.13	0.06	0.90	0.89	0.69	1.23
PEAQ20. El consumo de comida orgánica es una forma de proteger el planeta para las generaciones futuras.	−0.05	0.06	1.00	1.01	0.66	0.92
PEAQ21. Para mí es importante controlar la contaminación de mi carro.	−0.38	0.06	1.37	1.39	0.61	0.48
PEAQ23. El uso reducido de productos potencialmente tóxicos o fragancias para la limpieza en el hogar es una forma de preservar la naturaleza.	−0.09	0.06	1.08	1.05	0.65	0.85
PEAQ24. Reducir la contaminación de mi carro contribuye a la protección del medio ambiente.	−0.21	0.06	1.21	1.21	0.63	0.69
PEAQ25. El consumo de comida orgánica contribuye a reducir la aparición de cáncer.	0.2	0.06	1.00	0.98	0.65	1.04
PEAQ26. Una forma de contribuir a la conservación de los recursos energéticos es usar el transporte público tan frecuentemente como sea posible.	0.32	0.06	1.00	1.13	0.63	0.97
PEAQ27. Reducir mi consumo de agua ayuda a proteger los recursos naturales.	0.25	0.06	1.04	1.18	0.61	0.81
PEAQ28. Reducir el uso de productos potencialmente tóxicos de limpieza y fragancia en el hogar, contribuye a la protección del medio ambiente.	−0.07	0.06	1.04	1.00	0.66	0.97

The person ability range was −5.33–4.07 and the mean person ability was M = −0.30 logits; SD = 1.64. The item difficulty range was −0.38–0.34 and the mean item difficulty was M = 0; SD = 0.17.

### Reliability

The PEAQ reliability coefficients are Cronbach's alpha (α = 0.96), omega (Ω = 0.96) Rasch persons (0.90), and Rasch items (0.85). All reliability indexes are high (>0.80) indicating an adequate consistency in the scores of the scale. The people's ability separation is 3.03 and the items' difficulty is 2.42, which point to the existence of more than one level of ability and the item difficulty.

### Differential Item Functioning (DIF)

For the gender variable, we estimated the DIF for each item of PEAQ using the Mantel Haenszel test since it is the current standard due to the fact that does not confuse the real differences between the groups in the measure ability, it provides an estimate of the magnitude of the bias present in the item and a test of statistical significance (Table 3). The items PEAQ19, PEAQ21, and PEAQ28 exhibit a significant DIF (*p* < 0.05), nonetheless, those differences between groups are not relevant because the magnitudes obtained between men and women in the mentioned items (DIF contrast) are less than the criteria of 0.43 establish as negligible by the ETS (Zwick et al., 1999).

**Table 3:** Mantel Haenszel statistics (*p* < 0.05) of the PEAQ items with the gender bias variable (*N* = 415).

**Item**	**M, Women,** ***n* = 195**	**M, Men,** ***n* = 220**	**DIF contrast**	**SD^**af**^**
PEAQ19	0.00	0.28	−0.28	1.64
PEAQ21	−0.27	−0.51	0.25	
PEAQ28	0.05	−0.20	0.24	

*SD^af^, standard deviation of the ability in the factor that includes the item*.

We estimated DIF for each item of the PEAQ for education level using Mantel Haenszel (*p* < 0.05) but differences were not significant (*p* > 0.05) because the differences obtained between the educational levels in the mentioned items (DIF contrast) are less than half of the standard deviation of the scale (Table 4).

**Table 4:** Mantel Haenszel statistics (*p* < 0.05) of the PEAQ items with the educational level bias variable (*N* = 415).

**Item**	**Educational level**							
	**Elementary, *n* = 34**	**Secondary, *n* = 43**	**Technician, *n* = 67**	**Technologist, *n* = 74**	**Undergraduate, *n* = 113**	**Graduate,** ***n* = 84**	**DIF contrast**	**SD^**af**^**
PEAQ9	0.41		−0.18				0.58	1.64
PEAQ9	0.41			−0.32			0.73	1.64
PEAQ9	0.41				−0.18		0.58	1.64
PEAQ9	0.41					−0.39	0.80	1.64
PEAQ19	−0.07				0.32		−0.39	1.64
PEAQ19	−0.07					0.11	−0.17	1.64
PEAQ26	0.10					0.36	−0.27	1.64

*SD^af^, standard deviation of the ability in the factor that includes the item*.

### Criterion-Related Validity

The Spearman's rho correlations estimated between PEAQ scores and the EA (*r* = 0.859; *p* = 0.001), EV (r = 0.875; *p* = 0.001) and with PWQ (*r* = 0.885; *p* = 0.001) indicate a high and significant correlation.

## Discussion

The objective of this study was to assess the validity and reliability of the Colombian version of the PEAQ questionnaire among the employees working in organizations in the department of Huila.

The psychometric indicators obtained in this study for the PEAQ show that the scale scores allow inferences to be made about the pro-environmental attitudes of workers since support was found for the unidimensionality of the PEAQ through the values found of the explained variance of the measure 47.4% [above the criteria >40% (Linacre, 2006)], the variance not explained by the first residual 4.3% [below the criteria <20% (Reckase, 1979)] as well as an eigenvalue 1.67 [below the criteria <3 (Linacre, 2011; Bond and Fox, 2015)]. Furthermore, all statistical items fit the Rasch model, so these results also support the unidimensionality of the questionnaire (Bond and Fox, 2015; Boone and Staver, 2020). The reliability coefficients of the PEAQ are high: Cronbach's Alpha (α = 0.96) and omega (Ω = 0.96), and both are consistent with the results (α = 0.93) found by Félonneau and Becker (2008).

The PEAQ in this study person separation statistic (≥ 1.75) indicates that the PEAQ has more than two levels of ability (Bond and Fox, 2015; Boone and Staver, 2020). The same is true for the item separation statistic (2.42).

There is no evidence of DIF in the PEAQ items by gender and educational level, because none of the item presents a DIF greater 0.43, so the differences are considered insignificant (Zwick et al., 1999; Bond and Fox, 2015; Boone and Staver, 2020).

The correlation coefficient obtained as evidence of concurrent validity was significant and high with the three scales (EA, EV, and PEWQ) used as external criteria (Campbell and Fiske, 1959). This coincides with the evidence found in literature reviews (Lo et al., 2012; Young et al., 2013; McDonald, 2014; Inoue and Alfaro-Barrantes, 2015; Norton et al., 2015; Yuriev et al., 2020), which account for the relationship between these variables and the PEAQ.

One of the limitations of this study is that all respondents were volunteers, mainly from the education sector (higher education, technical, and technological), health (municipal entity providing low complexity health services), and other services (e.g., tourism and consulting). It is recommended to conduct a future study that includes a representative sample of different economic sectors. The results reflect that the variation range of the items' difficulty is low, which suggests that they have similar probabilities of endorsement by the persons of the sample. Close separation levels could be an indicator of the construct not being sufficiently measure in the sample, so additional items might be needed. Also, it is necessary to review and adjust the three items that were eliminated in this study for not meeting the Rasch model fit criteria, and conduct a new field study.

In spite of the above, the Colombian version of the PEAQ exhibits reliability, validity and invariance indicators, which support that the scores obtained with this instrument allow making adequate inferences about the pro-environmental attitudes of workers in Huila. Therefore, this version of the PEAQ has sufficient psychometric quality to be used in new research and organizational processes that seek to evaluate this construct in this population, with a view to developing baselines that allow the development of intervention processes to improve environmental sustainability in organizations, based on the analysis of individual behaviors, as is the case of pro-environmental attitudes.

The instrument can contribute to the evaluation of intervention programs implemented in organizations to, for example, generate energy savings, recycling, and reduction or non-use of polluting products. From this perspective, it is understood that it is in the employees where significant changes can be generated toward the sustainability of the organizations and the care of the environment, for which this instrument is a significant contribution.

## Data Availability Statement

The raw data supporting the conclusions of this article will be made available by the authors, without undue reservation.

## Ethics Statement

The studies involving human participants were reviewed and approved by Ethics Committee, Faculty of Psychology Universidad Católica de Colombia. The participants provided their written informed consent to participate in this study.

## Author Contributions

All authors listed have made a substantial, direct, and intellectual contribution to the work and approved it for publication.

## Conflict of Interest

The authors declare that the research was conducted in the absence of any commercial or financial relationships that could be construed as a potential conflict of interest. The handling editor ON declared a past co-authorship with the author WS.

## Publisher's Note

All claims expressed in this article are solely those of the authors and do not necessarily represent those of their affiliated organizations, or those of the publisher, the editors and the reviewers. Any product that may be evaluated in this article, or claim that may be made by its manufacturer, is not guaranteed or endorsed by the publisher.

## Footnotes

^1^Sierra-Barón, W. and Meneses Baez, A. L. (Forthcoming). *Content validity for a Pro-environmental behavior at work: internal and external factors questionnaire - Spanish version*.
